# A complete genome sequence for a phylogroup IV pea streak virus from alfalfa (*Medicago sativa* L.)

**DOI:** 10.1128/mra.00888-26

**Published:** 2026-10-15

**Authors:** Edison Reyes-Proano, Megan Perales, Erik J. Wenninger, Alexander V. Karasev

**Affiliations:** 1 Department of Entomology, Plant Pathology, and Nematology, University of Idaho 5640, Moscow, Idaho, USA; 2 Forage Genetics International 71381, Nampa, Idaho, USA; 3 Department of Entomology, Plant Pathology, and Nematology, Kimberly Research and Extension Center, University of Idaho 5640, Kimberly, Idaho, USA; Portland State University, Portland, Oregon, USA

**Keywords:** pea streak virus, carlavirus, new strain

## Abstract

We report the complete genome sequence of a phylogroup IV strain of pea streak virus (PeSV). The 8,013 nt, positive-strand RNA genome encodes five open reading frames characteristic of carlaviruses. This strain of PeSV from alfalfa (*Medicago sativa* L.) belongs to the newly identified phylogroup IV, which has not been previously reported in North America.

## ANNOUNCEMENT

Pea streak virus (PeSV) has filamentous particles and belongs to the genus *Carlavirus* in the family *Betaflexiviridae* (1). The virus has a single-stranded, positive-sense RNA genome of ca. 8.0 kb spanning five open reading frames (1). PeSV exists as a complex of strains forming at least six phylogroups (2, 3). PeSV is prevalent in several legume hosts in North America (4–7) and common in alfalfa in China (8). All 22 North American, field-collected PeSV isolates sequenced to date belonged to the same phylogroup I and exhibited very low genetic diversity, with 96–99% nt identity (3), representing different isolates of the same strain of PeSV. The global genetic diversity of PeSV, however, is high, with other phylogroups exhibiting only 75–78% nt identity relative to the North American phylogroup I (2, 3, 8).

In November 2025, leaf and stem tissues from a symptomatic alfalfa (*Medicago sativa* L.) plant displaying mosaic and yellowing were sampled in a greenhouse collection of breeding lines in Idaho. After grinding in liquid nitrogen, total RNA was extracted using the Spectrum Plant Total RNA kit (Sigma, St. Louis, MO, USA), libraries were prepared using the TruSeq Stranded Total RNA with Ribo-Zero Plant Kit (Illumina) and subjected to high-throughput sequencing (HTS) on a NovaSeqX+ platform. A total of 30,123,732 (2 × 150 bp) raw reads were produced, and 25,127,414 (2 × 150 bp) clean reads (PHRED score ≥15) were retained for the ALF02 sample. Raw reads were adapter and quality cleaned using Trimmomatic v0.39 (9) and mapped against the *M. truncatula* reference genome (GCF_003473485.1) using Bowtie2 v2.4.1 in local mode (10); 7,418,244 clean unmapped paired-end reads were subjected to assembly using SPAdes v3.15.3 in rna mode (11) and analyzed using the BLAST tools available in the National Center for Biotechnology Information (NCBI) package (12) against custom virus nucleotide and protein databases directly downloaded from the NCBI RefSeq database on 4 April 2024 using the query “Viruses.”

**Fig 1:**
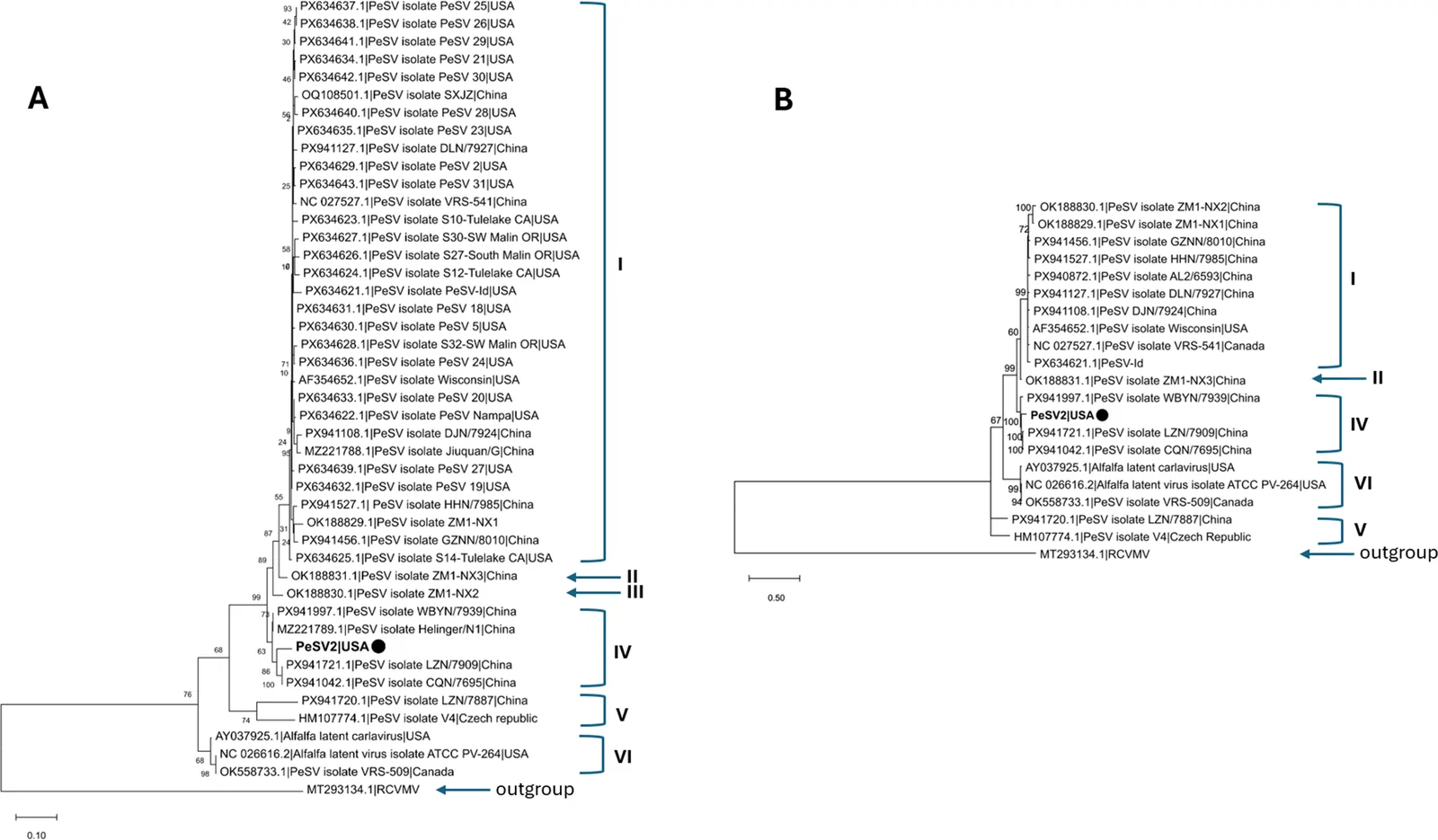
Phylogenetic relationships of the genetic variants of PeSV available in the GenBank database and the new PeSV2 sequence obtained in this work. Nucleotide sequences were aligned with the Clustal Omega 1.2.2 program in Geneious Prime 2026.1.2, with default settings. The best model fit was determined for each alignment using MEGA12. Phylogenies were constructed using maximum-likelihood method in MEGA12 (13) and 1,000 replicates. (**A**) Phylogenetic relationships based on the capsid protein gene of PeSV. The phylogenetic tree was inferred using the Hasegawa–Kishino–Yano substitution model following a gamma distribution (+G) with invariant sites (+I). (**B**) Phylogeny inferred from 19 genome sequences of PeSV from the main phylogroups of PeSV identified by Reyes-Proaño et al. (3) and the novel PeSV2, using the general time-reversible model following a gamma distribution (+G) and invariant sites (+I). The red clover vein mosaic virus genome (MT293134.1) was used as the outgroup. The PeSV2 sequence is shown in bold and indicated by a dark circle. Brackets identify PeSV phylogroups designated with Roman numerals from I to VI as described in reference 3.

The sequenced ALF02 sample yielded eight PeSV-specific contigs ranging from 258 to 1,491 nt in length, with an average sequencing depth of 2.08–7.02×, with the closest match (ca. 89.4–92.0% nt identity) to the sequence ZM1-NX3 (OK188831) from China based on the BLASTn program (NCBI). The same PeSV-specific contigs from the ALF02 sample, named PeSV2, displayed only 85.4–85.6% identity to the isolate PeSV-Id (PX634621) from an Idaho field alfalfa. To extend the genome sequence for PeSV2, additional primers (Table 1) were used in RT-PCR as described in reference 3 to amplify virus-specific sequences between the HTS-derived contigs, assembly was then verified by Sanger sequencing and by remapping the HTS reads to it. The 5′ and 3′ ends of the PeSV genome were acquired using specific primers (Table 1) and the 5′/3′ RACE Kit (Roche, Indianapolis, IN, USA) as described in reference 3. The sequenced PeSV2 genome of 8,013 nt (GenBank accession PZ713317) was 93.9% identical to a Chinese PeSV sequence (PX941721) in a pairwise comparison by BLASTn, had a 42.8% GC content (Geneious Prime 2026.1.2, Biomatters), and was covered by 698 reads. In a phylogenetic analysis (Fig. 1), PeSV2 was placed in a new phylogroup IV not previously reported in North America.

**TABLE 1:** Primers used to confirm (C) the presence, extend (E) sequences, and acquire 5′ (A5) and 3′ (A3) termini of the PeSV sequence under study (PeSV2)^*a*^

Primer name	Genome position	Oligonucleotide sequence (5′ to 3′)	Intended use
PeSV2-3F	1112	AATAGTACAAGAGCCCAGCGC	C
PeSV2-3R	1610	GTAAAAGCTGTAGGGATTCAATTCC	
PeSV2-4F	1612	ATTTGCTAGTAGAGAGGGCAGAAGG	C
PeSV2-4R	2073	CAAAACCATTACACTCCAACAACAT	
PeSV2-5F	2293	ACCATAAGCACGCTGTGAGC	C
PeSV2-5R	2774	GCACTGCCTTAAATCATTCTTCG	
PeSV2-6F	2859	GGGAAGACTTTGCAAGATATTCATAGG	E
PeSV2-6R	3644	ATATGTTACACAATCCAAATACCCTGG	
PeSV2-9F	4902	ATTTGCACTAAGTTCGACAACAGG	E
PeSV2-9R	5695	CAATTGTAAAAAGCATCAGTCTCTTCC	
PeSV2-10F	5669	GGAAGAGACTGATGCTTTTTACAATTG	E
PeSV2-10R	6099	CTTTTCCCAATCACCTCTCTGG	
PeSV2-11F	6604	CATTATATTCACTTTGAGATCCAGCC	E
PeSV2-11R	7043	AAACTTAAAGATTCTCAAATCGTTTGC	
PeSV2-12F	6929	GTACCATCATTCTTACTGGTGAGAGTG	C, E
PeSV2-12R	7731	CGAATGCAGCAAACTTGACAT	
PeSV2_5-1R	555	GCTTGCTTGAAAATTGAGG	A5
PeSV2_5-2R	490	AAAGTTCTTCTGATTGTGAACTCCG	A5
PeSV2_5-3R	452	TTTGTCAGCACTGGTGACATACC	A5
PeSV2_5-4R	261	CTGAAAAGGGACTGAGATACAAACC	A5
PeSV2_3-1F	7598	GGAACAGTGCACTCTGAGG	A3
PeSV2_3-2F	7711	ATGTCAAGTTTGCTGCATTCG	A3
PeSV2_3-3F	7757	AGCATCTATTCAACCCGCG	A3

^*a*^ The 5′ and 3′ ends of PeSV genome were acquired using specific primers and the 5′/3′ RACE Kit (Roche, Indianapolis, IN) as described in reference 3.

## Data Availability

The complete coding genome sequence of the PeSV2 isolate is available in GenBank under accession number PZ713317. The raw sequence data SRR39693583 were deposited in the NCBI Sequence Read Archive under BioProject accession no. PRJNA1497020.
